# The Gut Microbiome Advances Precision Medicine and Diagnostics for Inflammatory Bowel Diseases

**DOI:** 10.3390/ijms252011259

**Published:** 2024-10-19

**Authors:** Walaa K. Mousa, Aya Al Ali

**Affiliations:** 1 College of Pharmacy, Al Ain University of Science and Technology, Abu Dhabi 64141, United Arab Emirates; aya.alali@aau.ac.ae; 2 College of Pharmacy, Mansoura University, Mansoura 35516, Egypt; 3 AAU Health and Biomedical Research Center, Al Ain University, Abu Dhabi 112612, United Arab Emirates

**Keywords:** gut microbiota, precision medicine, live therapeutics, diagnostics, microbiome editing, IBD

## Abstract

The gut microbiome emerges as an integral component of precision medicine because of its signature variability among individuals and its plasticity, which enables personalized therapeutic interventions, especially when integrated with other multiomics data. This promise is further fueled by advances in next-generation sequencing and metabolomics, which allow in-depth high-precision profiling of microbiome communities, their genetic contents, and secreted chemistry. This knowledge has advanced our understanding of our microbial partners, their interaction with cellular targets, and their implication in human conditions such as inflammatory bowel disease (IBD). This explosion of microbiome data inspired the development of next-generation therapeutics for treating IBD that depend on manipulating the gut microbiome by diet modulation or using live products as therapeutics. The current landscape of artificial microbiome therapeutics is not limited to probiotics and fecal transplants but has expanded to include community consortia, engineered probiotics, and defined metabolites, bypassing several limitations that hindered rapid progress in this field such as safety and regulatory issues. More integrated research will reveal new therapeutic targets such as enzymes or receptors mediating interactions between microbiota-secreted molecules that drive or modulate diseases. With the shift toward precision medicine and the enhanced integration of host genetics and polymorphism in treatment regimes, the following key questions emerge: How can we effectively implement microbiomics to further personalize the treatment of diseases like IBD, leveraging proven and validated microbiome links? Can we modulate the microbiome to manage IBD by altering the host immune response? In this review, we discuss recent advances in understanding the mechanism underpinning the role of gut microbes in driving or preventing IBD. We highlight developed targeted approaches to reverse dysbiosis through precision editing of the microbiome. We analyze limitations and opportunities while defining the specific clinical niche for this innovative therapeutic modality for the treatment, prevention, and diagnosis of IBD and its potential implication in precision medicine.

## 1. Introduction

The human body is colonized by trillions of microbial species, their genes, and secreted metabolites, collectively known as the human microbiome [1]. The gut microbes exceed 95% of the entire human microbiota, representing the most colonized microbial ecosystem in the body [2,3]. This dynamic microbial community provides a plethora of metabolic functions and molecular signaling crucial to host health. One of the fundamental functions of gut microbes is the maturation of the gut immune system and the maintenance of local immune homeostasis. While a balanced microbial community is crucial for gut health, a disruption in this balance, known as dysbiosis, is linked to a broad range of diseases such as IBD [4], a condition characterized by chronic inflammation of the GI tract, leading to symptoms such as abdominal pain, severe diarrhea, fatigue, weight loss, and malnutrition. IBD is at the focal point of microbiome research, although its heterogenicity makes it a challenging task to understand how microbiome dysbiosis fully affects disease etiology and progression [5]. Various studies revealed that the gut microbial community in IBD lacks specific microbes such as *Faecalibacterium prausnitzi*, *Akkermansia muciniphila*, *Clostridium buytricum*, *Roseburia*, *Bifidobacterium*, and *Lactobacillus* [6,7,8]. Further research has provided mechanistic insights into how these microbes contribute to gut health. For example, these microbes help by producing anti-inflammatory metabolites such as butyrate. Another action is balancing the host’s immune response through the induction of Treg cells and anti-inflammatory cytokines. In addition, gut microbes are crucial in maintaining the integrity of the gut mucosal barrier, preventing the leakage of toxins and pro-inflammatory metabolites [8,9].

Given the crucial role of gut microbes in human health and, in particular, local inflammatory conditions in the gut, there has been a significant increase in interest in modulating the gut microbiome using either live or synthetic products or by changing diet regimes. As more individualized interactions, functions, and phenotypes of gut microbiome emerged, it became a target for new therapeutics and diagnostics that advance precision medicine, especially when combined with other multiomics data [10]. Emerging research is on the modulation of the microbiome to manage IBD by altering the host immune response. Another interesting advance is the development of targeted approaches to reverse dysbiosis through precision editing of the gut microbial community. In this review, we discuss and summarize recent knowledge of the role of the gut microbiome in the progression and therapy outcomes in IBD. We analyze advances in developing microbiome-based therapeutics and diagnostic biomarkers and discuss gaps and challenges in the field.

### 1.1. Early Interaction Between Gut Microbes and the Immune System Is Essential for Its Maturation and Robustness

Our understanding of how the immune system tolerates or ignores trillions of microbial cells, that exceed the count of human cells, is advancing [11,12]. It is reasonable that the immune system has evolved to tolerate commensal microbes to retain their unreplaceable functions, eventually leading to mutual dependence. Microbes in the gut are sensed by the host immune system mainly through dendritic cells (DCs) and Toll-Like Receptors (TLRs) [13]. The binding of microbes in the gut lumen, or those adherents to the outer mucosal layer to TLRs expressed on gut epithelial cells, stimulates DCs to penetrate the intestinal epithelial cells (IECs) through the tight junctions and sample the microbes [13,14]. Thereafter, DCs carry antigens of microbes representing them to host immune cells, leading to immune tolerance [14].

**Figure 1:**
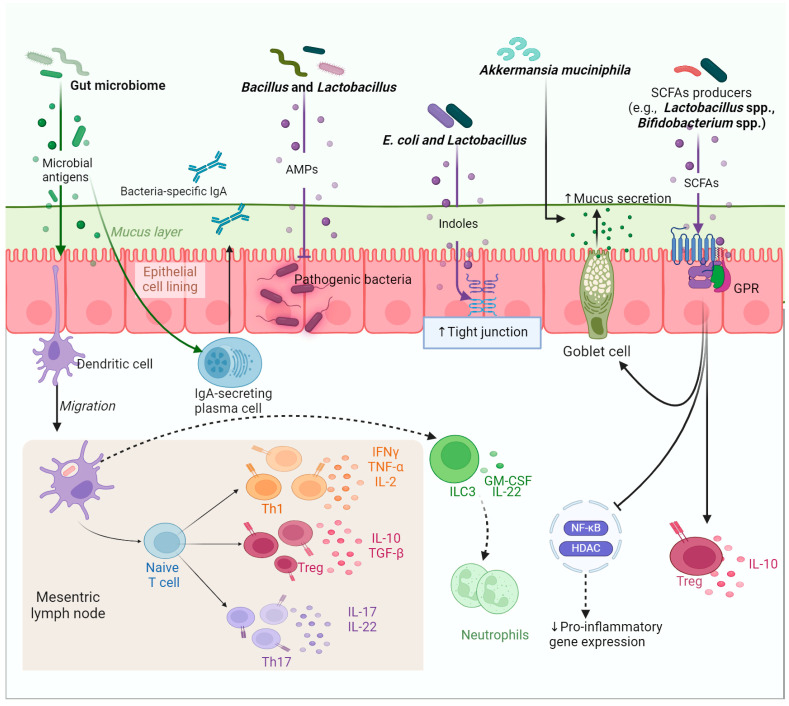
Role of the microbiome in intestinal homeostasis. The gut microbiome contributes to the maturation of the gut immune system. Microbial antigens are detected by dendritic cells, which migrate to the mesenteric lymph nodes and prime naïve T cells into specialized subtypes such as Th1, Th17, and Treg cells. These T cells are the main sources of various cytokines like the anti-inflammatory IL-10, which helps prevent inflammation, and the pro-inflammatory IL-17, which promotes rapid pathogen defense. Moreover, bacterial antigens, such as lipopolysaccharides, activate ILC3 cells, inducing the release of GM-CSF and IL-22, which recruit neutrophils for further pathogen protection. Moreover, the gut microbiome primes B cells into IgA-secreting cells. Secreted IgA provides an additional way of defense. Many microbiome species are known to produce anti-microbial peptides (AMPs) that directly inhibit harmful bacteria in the gut. Commensal metabolites also contribute to gut homeostasis and enhance gut barrier function by increasing the expression of tight junction proteins, while short-chain fatty acids (SCFAs) bind to G protein-coupled receptors (GPRs), promoting anti-inflammatory activity. SCFAs directly induce Treg cells to produce IL-10 and block NF- κB signaling and histone deacetylase (HDAC) activity, thus reducing the expression of pro-inflammatory genes. In addition, SCFA-producing bacteria and *Akkermansia muciniphila* promote the production of mucin, a main component in the mucus layer.

Gut microbes are essential for the development and maturation of mucosal immunity and the maintenance of gut immune homeostasis [13]. Examples of the mechanisms by which gut microbes contribute to the health of the gut are illustrated in Figure 1. The main component of the mucosal immune system is the gut-associated lymphoid tissues (GALTs), which are responsible for detecting and responding to antigens in the gastrointestinal tract, distinguishing between harmful and tolerable or self-antigens [15]. In addition to their role in regulating the mucosal immune response in homeostasis and inflammatory diseases such as IBD, GALTs consist of an integrated network of tissues including (1) Peyer’s Patches, located in the ileum, which are clusters of lymphoid follicles rich in B and T lymphocytes, (2) Lamina Propria, a connective tissue layer under the epithelium contains macrophages and dendritic cells; (3) Intraepithelial Lymphocytes, T cells within the intestinal epithelium that help maintain barrier integrity and regulate inflammation; and (4) Mesenteric Lymph Nodes in the surrounding connective tissue, which filter lymphatic fluid and coordinate immune responses [16]. Microbial colonization of the gut soon after birth is essential for the proper development and maturation of GALT. They stimulate the growth and differentiation of various immune cells within the GALT, including T cells, B cells, and antigen-presenting cells, by exposing them to microbial antigens. This exposure helps in the training and maturation of the immune response, enabling a more effective defense against pathogens while maintaining tolerance to non-threatening antigens of commensal microbes. Gut microbes modulate GALTs via epigenetic signals to enhance their tolerance.

### 1.2. Gut Microbes Are Responsible for the Fine-Tuning of the Local Immune Response

Maintaining cytokine balance, mainly by maintaining Treg cells and Th1/Th17 at a steady state, is critical to regulating mucosal homeostasis [17]. Some bacteria such as *F. prauznitsii* induce Tregs differentiation, resulting in enhanced production of the anti-inflammatory cytokine, IL-10 [14]. Other gut microbes stimulate the production of IgA, which protects from invading pathogens while inducing the proliferation of Treg cells and enhancing the production of IL-10 to suppress inflammation [18].

Gut microbes interact with immune cells through their secreted metabolites such as short-chain fatty acids (SCFAs), which modulate inflammation. For example, butyrate is SCFA produced by commensals such as *Bacteroides thetaiotaomicron* and *F. prausnitzii* [19], which exerts an anti-inflammatory effect through multiple mechanisms including the induction of Treg cell differentiation and the production of anti-inflammatory cytokines such as IL-10 and IL-18 [20,21]. A reduction in fecal SCFAs is common in IBD [22]. Butyrate functions by binding to G protein-coupled receptors (GPCRs) and as a histone deacetylase (HDAC) inhibitor [20]. Other bacterial metabolites implicated in the immune response are bile acid metabolites that can control the expression of genes related to T helper cell differentiation [23]. Some gut microbes convert lithocholic acid into isolithocholic acid (isoLCA) and 3-oxolithocholic acid (3-oxoLCA) via the enzymatic activity of the 3α-hydroxysteroid dehydrogenase enzyme [23]. Both of these bile acid metabolites block retinoic acid receptor-related orphan nuclear receptor-γt, which leads to the suppression of Th17 differentiation and a corresponding reduction in the proinflammatory interleukin, IL-17 [23]. Interestingly, the gene encoding the 3α-hydroxysteroid dehydrogenase enzyme in IBD patients was found to be significantly under-expressed [23]. Tryptophan metabolites are microbial products that interact and activate aryl hydrocarbon receptor (AhR), inducing CD4+ T cells and innate lymphoid cells within the gut [24]. Patients with IBD display diminished production of microbiome-derived AhR ligands because of the altered microbiome composition.

### 1.3. Gut Microbes Maintain Homeostasis by Enhancing the Integrity of the Mucosal Barrier

One of the strategies evolved by gut microbes to modulate local mucosal immunity and maintain homeostasis is maintaining the integrity of the mucosal barrier and its function [13]. Data from germ-free (GF) mice show that inoculation with *B. thetaiotaomicron* modulates the expression of several genes including those implicated in mucosal barrier fortification, postnatal intestinal maturation, and xenobiotic degradation [25]. Microbes stimulate the production of mucin from the intestinal goblet cells. Mucin forms a protective layer on top of gastrointestinal cells, acting as a barrier to minimize direct exposure to microbes or their metabolites, ultimately decreasing inflammation [26]. Microbial metabolites such as butyrate promote mucin production and stimulate the growth and repair of the colonocytes lining the colon. A study showed that bacteria can penetrate the IECs of mucin-deficient mice, causing inflammation and cancer [27]. Further, mucin concentrates microbial metabolites, facilitating microbe–host communication. Gut microbes induce the expression of tight junction proteins, preventing leakage of metabolites and microbes from the gut into the systemic circulation, which otherwise may result in systemic inflammation [28]. In addition, some gut microbes such as *Escherichia coli* metabolize tryptophan to indole derivatives, which promote the development of tight junctions in the intestine and reduce the epithelial layer permeability [29,30]. The ability of gut microbes to compete with invading pathogens also contributes to the integrity of the barrier, which otherwise will be exposed to disruption and damage by pathogens. Gut microbes stimulate the production of host-antimicrobial peptides, which are sequestered from the gut lumen and absorbed deep inside the mucosal layer, making it almost microbe-free [31].

### 1.4. Gut Microbes Modulate the Host Immune Response via microRNAs (miRNAs)

**Figure 2:**
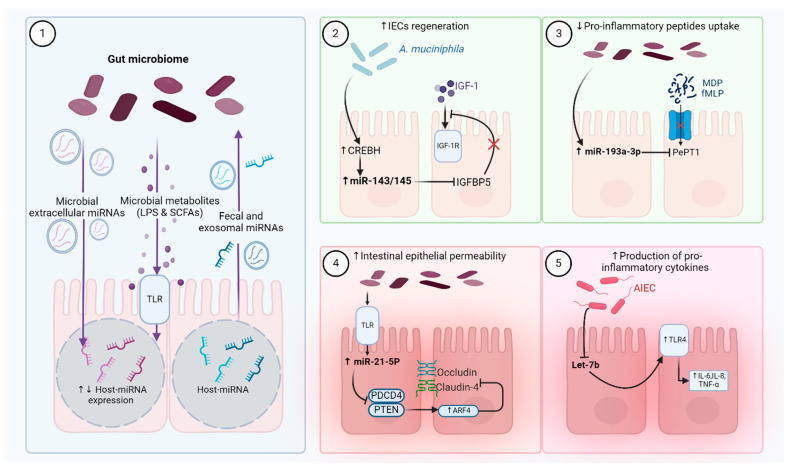
(**1**) The bi-directional relationship between the host and microbe miRNAs. Gut microbiota miRNAs are secreted in extracellular vesicles (EVs) that regulate host gene expression through complementary binding to host mRNA. Moreover, microbe-derived metabolites and bacterial components can also influence host miRNA expression mainly through the toll-like receptor (TLR)/MyD88 pathway. On the other hand, host-released miRNAs in EVs and feces are taken by the gut microbiome, which alters their abundance, function, and growth. (2,3) Role of the microbiome–miRNA axis against IBD. (**2**) *A. muciniphila* supports the regeneration of intestinal epithelial cells (IECs) by stimulating cAMP-responsive element-binding protein H (CREBH), a transcription factor known for its inhibitory activity against inflammatory responses. The elevation in CREBH expression leads to the upregulation of host miR-143 and miR-145, which activate insulin-like growth factor (IGF), a stimulator of intestinal proliferation and injury recovery, by suppressing the endogenous suppressor of IGF, IGFBP5. (**3**) Host- derived miR-193a-3p also prevent inflammation. miR-193a-3p inhibits the activity of the transporter PepT1. Through this inhibition, no passing of bacterial products that promote inflammation, such as N-Formyl-Methionyl-Leucyl-Phenylalanine (fMLF) or muramyl dipeptide (MDP), occurs. (**4**,**5**) Role of the microbiome–miRNAs axis in promoting IBD. (**4**) Microbial stimulation of toll-like receptors (TLRs) upregulates the expression of miR-21-5P in IECs. miR-21-5P suppress phosphatase and tensin homolog (PTEN) and programmed cell death 4 (PDCD4)l this suppression upregulates ADP ribosylation factor 4 (ARF4), a GTPase that inhibits tight junction-related proteins claudin-4 and occluding, thus elevating intestinal permeability. (**5**) Adherent invasive *Escherichia coli* (AIEC) suppresses the expression of let-7b in the settings of Crohn’s disease. This activates TLR4, which elevates pro-inflammatory cytokines release, promoting mucosal inflammation and immune attacks against gut microbiota.

Gut microbes can modulate the host immune response through miRNAs, as shown in Figure 2. For example, a study showed that mice with colitis have a reduced expression of miRNA-10a that corresponds with a higher level of pro-inflammatory cytokines such as IL-12/IL-23p40 [32]. Further, microbial inhibition of miR-375-3p promotes the proliferation of IECs [33], while microbial stimulation of miR-21-5p increases the permeability of IECs, leading to inflammation [34]. The effects of commensal microbes on host gene expression could also be mediated by microbial metabolites such as lipopolysaccharides (LPSs) and SCFAs [35]. A study reported that butyrate changes the expression of 44 miRNAs in HCT-116 colon cancer cells [35]. For example, miR-106b affects the expression of the p21 gene which mediates the anti-inflammatory effect of microbial SCFAs [35]. Maintaining control over host miRNAs is crucial to regulate intestinal barrier function, affecting susceptibility to inflammatory diseases. GF mice show significant differential expression in 16 miRNA genes when compared with conventional mice [36]. These genes mostly regulate immune function and intestinal homeostasis through the regulation of the intestinal barrier [36]. *A. muciniphila* and its membrane protein Amuc_1100 promote intestinal epithelial cell regeneration and support barrier function by upregulating the expression of miR-143/145 in the colon, which modulates the insulin-like growth factor-1 (IGF-1) signaling pathway [37]. Some commensals such as *L. salivarius* and *L. fermentum* exhibit anti-inflammatory activity by enhancing the expression of miRNA-150 and miRNA-143 in a mouse model of colitis [38]. Other microbial foes such as *Fusobacterium nucleatum* increase resistance to chemotherapeutics by decreasing the expression of miRNA-18a and miRNA-4802, resulting in interference in autophagy pathways [2]. Other studies reported that *F. nucleatum* can inhibit the anti-tumor T cell response, leading to the progression of cancer through a modulatory effect on miRNA-21, which increases the level of prostaglandin E2 and IL-10; however, the exact mechanism is not clear [39]. In addition, *F. nucleatum* employs miRNA to stimulate the expression of the nuclear factor kappa B (NF-κB) gene, resulting in inflammation [39]. In contrast, *F. prausnitzii* silences NF-κB gene expression through hyperacetylation [40]. Some species of *E. coli* are implicated in causing colorectal cancer (CRC) by inducing the expression of miR-20a-5p, which enhances the expression of some growth factors leading to cancer [41]. Although the precise mechanism is not clearly understood, some data suggest a possible role of colibactin, a microbial metabolite encoded in *E. coli* Nissle 1917, in developing CRC by directly damaging DNA [41]. A recent study found that colibactin can awaken latent bacteriophage in response to DNA damage resulting in indirect and unique lethal action against gut microbes, which consequently affect gut microbes’ structure and function [42]. Several miRNAs control cellular functions such as the immune response by the direct control of immune cell differentiation, while abnormal miRNAs are linked to autoimmune diseases [43]. For example, commensal microbes can downregulate the expression of miR-10a on dendritic cells targeting IL-12/IL-23p40, contributing to immune homeostasis [32]. Some microbial infections are noted to affect miRNA expression, leading to changes in the host immune response and predisposition to diseases. For example, *Mycobacterium tuberculosis* decreases miR-let 7f in infected macrophages, leading to a decrease in the production of tumor necrosis factor (TNF) and IL-1β which suppress the immune system by affecting NF-κB inflammatory response [44]. A similar effect was noted for *Helicobacter pyroli* [45].

Several miRNAs are known to control gut microbes and intestinal homeostasis by directly modulating the expression of Treg cells and T helper cells such as Th1, Th2, and Th17 [46]. Th1 is claimed to drive Crohn’s disease, while Th2 induces ulcerative colitis and Th17 is more implicated in multiple sclerosis [46]. The differentiation of naïve CD4+ T cells into Th17 and Treg is under tight control by the host, gut microbes, microbial metabolites, and miRNA. Th17/Treg imbalance is a major driver of autoimmune diseases, particularly gastric inflammation and multiple sclerosis (MS) [46,47]. A study identified miRNA-141 and miRNA-200a as inducers for Th17 differentiation and repressors for Treg, leading to the progression of MS [48]. The study suggested that miRNA-141 and miRNA-200a might inhibit the repressor regulatory proteins for Th17 differentiation, leading to the overproduction of Th17 [48]. Another example is miRNA-155, which regulates Th17/Treg balance through TLRs [47]. The overexpression of miR-155 enhances Th17 immunogenic function and suppresses Treg cells, while the knockdown of miR-155 results in less inflammation [49]. The same effect is also noted for some gut microbes or their LPSs. A study showed that *L. salivarius* and *L. fermentum* restore normal levels of miR-155, maintaining the Th17/Treg balance and decreasing colitis-associated inflammation in mice mainly by restoring the gut barrier function [38]. Other examples of miRNA modulating gut immunity include miR-18b, miR-363-3p, and miR-106a [50]. These miRNAs suppress the differentiation of Th17 and the subsequent production of the pro-inflammatory interleukin IL17 [50]. A computational analysis aimed to predict the effect of 64 miRNA on Th17 based on their interactions with gene transcripts identified 11 miRNA that can modulate Th17 differentiation both as suppressors and inducers [51]. Inducer miRNA included miR-1, miR-27a, miR-27b, miR-30c, and miR-141, while repressor miRNA included miR-20a, miR-20b, miR-21, miR-93, miR-106a, and miR-152 [51]. Much interest is growing in identifying microbes or miRNAs that suppress Th17 overproduction and revert the shifted balance to decrease autoimmune flares. Controlling the interplay between miRNA and microbes seems to be an exciting development and intervention in the treatment or prevention of inflammatory and autoimmune diseases [52].

### 1.5. Microbial Dysbiosis in the Gut Disturbs Homeostasis and Drives IBD

**Figure 3:**
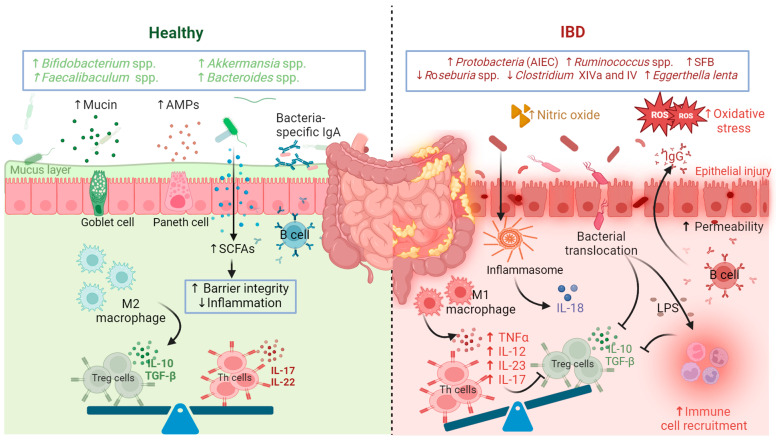
Structural and functional differences in intestinal mucosa between IBD patients and healthy individuals. During homeostasis (**left**), the gut maintains an eubiosis state represented by richness and diversity in commensal bacteria such as *Bifidobacterium* spp., *Akkermansia* spp., etc. This healthy state is supported by the immune system, in which a balance between Th cells, Treg cells, and their associated cytokines is maintained. In a healthy gut, M2 macrophages, known for their anti-inflammatory roles, are the predominant macrophage phenotype. Resistance against pathogens is also maintained by the immune system through B cells, which secrete IgA antibodies that specifically target harmful bacteria while skipping the resident microbiome. Moreover, the mucus layer serves as a protective barrier. Bacterial metabolites, such as short-chain fatty acids (SCFAs), help maintain an intact gut barrier, while other commensal species stimulate the production of mucin, the main protective component of the mucosal layer. Meanwhile, in IBD patients (**right**), perturbations in the gut microbiome community lead to a state of dysbiosis, with many SCFA producers depleted, while other inflammatory promoters are increased. Because of this imbalance, higher levels of inflammatory cytokines are secreted, exacerbated by the loss of mucus and the impairment in the mucosal layer integrity, which facilitate bacteria to translocate and further recruit immune cells like neutrophils while suppressing the anti-inflammatory function of Treg cells. Furthermore, M1 macrophages, which are often associated with gut inflammation, become more prevalent and secrete inflammatory cytokines. In contrast to a healthy gut, elevated levels of reactive oxygen species (ROS) and other mediators of intestinal inflammation, such as nitric oxide, both of which exacerbate inflammation, characterize the inflamed gut.

Microbiome dysbiosis disturbs the delicate immune balance, resulting in autoimmune and inflammatory diseases, as illustrated in Figure 3 [53,54]. IBD is a heterogeneous disease with different etiologies that vary with the location within the gastrointestinal tract and include Crohn’s disease (CD), ulcerative colitis (UC), and microscopic colitis (MC) [55,56]. Several studies confirmed the fundamental role of gut microbes in IBD [57]. Variations in the microbial makeup induce different levels of pro-inflammatory and anti-inflammatory mediators that might flare up IBD or keep it checked. For example, multiple studies reported a sharp reduction in *F. prausnitzii* in IBD patients [58]. *F. prausnitzii* is a known producer of butyrate and other proteins that inhibit the production of pro-inflammatory cytokines [6]. Another study reported that healthy controls with genetic risk factors for developing IBD have significantly altered microbiota composition characterized by a reduction in *Roseburia* genera [59], which converts acetate to butyrate. Segmented filamentous bacterium (SFB) induces Th17 differentiation, leading to excessive production of IL-17 and IL-22 [60]. Overall, this effect results in a higher level of inflammation and a stronger immune response in mice [60]. In addition, the reduction in SFB is linked to fewer Th17 and, subsequently, lower levels of IL-17 [61], while a higher abundance of SFB triggers autoimmune diseases such as rheumatoid arthritis and results in the production of autoantibodies [62]. DNAs derived from some bacteria in the small intestine suppress Treg differentiation through the stimulation of TLR9, which disrupts intestinal homeostasis [63]. Microbial dysbiosis drives leakages of microbial toxins and metabolites such as LPSs that cause inflammation and hyperactivity of the immune system [64].

Several studies revealed that CD patients, in particular, had lower microbial richness compared with UC patients. Both conditions exhibited a higher abundance of *Firmicutes* and *Actinobacteria*, while *Enterobacteriaceae* was increased in CD but lower levels were detected in UC [65]. In a study that used gene-level metagenomic mapping to identify diagnostic microbiome signatures [66], *Solobacterium moorei F0204* was identified as diagnostic for inflammatory bowel diseases [66]. Further data showed that CD patients have enrichment in adherent-invasive *E. coli* (AIEC), which is claimed to induce inflammation by irritating the gut lining [67]. AIEC also produces propionates, stimulating the production of IL-1β, a component of inflammasomes that increases the production of IL-18, a pro-inflammatory interleukin. An inflammasome is a multi-protein complex that increases the production of pro-inflammatory cytokines, resulting in intense inflammation [68]. A recent study showed that the administration of genetically engineered AIEC, which lacks an enzyme required for the synthesis of propionate, resulted in a low level of inflammation in mice with Crohn’s-like symptoms [69]. Another study reported that AIEC mediated a strong immune reaction and inflammation in CD patients by inhibiting the expression of the miRNA let-7b, resulting in the overproduction of pro-inflammatory cytokines [70].

### 1.6. Abnormal Expression of Host-Derived miRNAs Drives IBD Through the Manipulation of Gut Microbial Composition

The use of miRNA is one possible mechanism by which host genetics influence microbial population dynamics and their gene expressions, driving either a balanced or shifted microbiome [71]. Abnormal miRNA expression changes the abundance of microbes and their metabolites, which consequently results in diseases [46,71]. Fecal miRNA can even be used as a biomarker for microbial fluctuation with the onset and progression of gut diseases such as IBD and CRC [4]. A study showed that mice deficient in the production of miRNA had abnormal growth of gut microbes associated with a higher incidence of gut inflammation [52]. Interestingly, fecal supplementation with miRNA from wild-type mice to diseased mice restored the normal microflora and improved the inflammation symptoms [52]. Furthermore, miRNA secreted by gut epithelial cells affects the expression of genes related to the mucosal barrier, which subsequently affects microbial colonization [46]. Several examples of specific miRNAs controlling microbial abundance and subsequent host functions are noted [46]. The growth of *F. nucleatum* and *E. coli*, two bacterial species implicated in IBD and CRC, are upregulated by miR-515-5p and miR-1226-5p [2,4]. miR-21, an overly expressed miRNA in inflammatory bowel disease, escalated inflammation in an animal model of colitis through a possible control of gut microbes [72]. Aside from the direct control of microbial growth, some miRNAs affect the ability of intestinal cells to absorb microbial metabolites. For example, miR-193a-3p decreases the ability of intestinal cells to absorb microbial pro-inflammatory tripeptides (L-Ala-γ-D-Glu-meso-DAP), which otherwise cause inflammation [73]. miR-193a-3p exerts its effect by silencing the expression of PepT1 that is involved in facilitating the absorption of this metabolite [74] Interestingly, PepT1 is overexpressed in colitis, and data show that antibiotic treatments abolish its effect because of a lack of microbial products [73].

### 1.7. Restoring a Balanced Gut Microbial Composition to Manage IBD

Restoring the balanced composition of the gut microbial community to manage diseases such as IBD is gaining interest as an ecological therapy. One possible intervention to reverse dysbiosis and control IBD is supplementation or diet modulation that enriches the abundance of SCFA producers [75]. For example, some diet protocols restrict food implicated in inflammation to control IBD [76]. The diet is based on three phases as follows: (1) the elimination of specific food components such as gluten, refined sugar, eggs, dairy, coffee, and alcohol, (2) a maintenance phase until an observable improvement in the inflammation biomarkers is reached, and (3) the slow reintroduction of each food group [76]. Recently, a study reported that a diet deficient in fiber promoted the growth of mucolytic bacteria. The breakdown of the mucus protective layer triggered intestinal inflammation by boosting Th1 immune responses, altering the pattern of bacterial IgA coating, and increasing NK cell levels. This effect was particularly evident in genetically susceptible individuals, as demonstrated in mice lacking interleukin-10 [77].

Another approach is the development of microbiome-based therapeutics such as probiotics, prebiotics, fecal microbiota transplantation, or engineered bacteria. These therapies aim to reduce inflammation, repair the intestinal barrier, and normalize immune responses. As research advances, microbiome-based treatments hold the potential to offer more targeted and effective management options for individuals suffering from IBD. This alternative is also favorable compared with current IBD treatments such as corticosteroids and immunosuppressants, which are associated with long-term side effects.

## 2. Probiotics

Probiotics are living microorganisms that, when consumed in sufficient quantities, have the potential to yield favorable health effects [78]. Several human studies and animal models show the promise of using probiotics in managing IBD (Table 1), mainly by modulating the immune and inflammatory response by enriching SCFAs and diminishing pro-inflammatory cytokines [79]. Preliminary results show that probiotics ameliorate symptoms such as abdominal pain, diarrhea, and weight loss [80,81]. However, further clinical trials reported controversial results [82]. Interestingly, the same probiotics do not show comparable results in UC and CD [83]. For example, the *L. johnsonii* LA1 probiotic failed to prevent endoscopic recurrence in CD patients, although it was effective in UC [84]. In contrast, a probiotic mixture composed of *Bifidobacterium*, *lactobacillus*, and *enterococcus* regulated cognitive reactivity to the sad mood in CD patients and improved Leiden Index of Depression Sensitivity, but these effects were not observed in UC patients [85].

*E. coli* Nissle 1917 (EcN) is one of the commonly used probiotic in managing IBD and is still manufactured by the German pharmaceutical company Ardeypharm GmbH under the name Mutaflor^®^ [86]. This strain demonstrates safety and efficacy in sustaining remission among UC patients. These findings are comparable to those observed with mesalazine [87], which has been recommended as an effective alternative to maintain UC remission. Animal models and cohort studies showed that EcN restores normal levels of secretory IgA, promotes the production of host antimicrobial peptides, and reduces levels of IL-13, IL-5, TNF-α, and IFN-γ l [88]. Furthermore, EcN reverses the abnormal miRNA expression involved in the inflammatory response, including the upregulation of miR-155, miR-223, and miR-150 in colonic tissue [89].

Members of the gut-predominant Gram-positive genus *Bifidobacterium* have been widely investigated for their efficacy in targeting IBD. For example, an 8-week administration of *B. longum* 536 resulted in a reduction in UC disease activity index (UCDAI) and endoscopic index (EI) scores [90]. The oral intake of *B. longum* 51A in dextran sodium sulfate (DSS)-induced mice resulted in lower myeloperoxidase, and eosinophil peroxidase in colon [91]. Both enzymes catalyze the production of reactive oxygen species, which promote mucosal inflammation and damage. Similar effects were reported for *B. longum* YS108R and *B. animalis* subsp. *lactis* BB12 [92]. Further studies reported that *B. longum* Bif10 and Bif16 decreased the levels of TNF-α, IL-1β, IL-6, and IL-1, prevented epithelial damage, promoted the intestinal barrier function, and increased SCFAs levels [93]. In line with these findings, *B. longum* CECT 7894 can enhance the efficacy of infliximab, an anti-tumor necrosis factor agent commonly employed in IBD treatment. This positive effect is mediated by preventing goblet cell loss, reducing inflammatory cell infiltration in the colon tissue, and modulating the abundance of genera that express bile salt hydrolases and 7α-dehydroxylases genes, thus elevating fecal levels of secondary bile acids [94].

Multiple species of the *Lactobacillus* genus have demonstrated positive outcomes in targeting IBD-associated inflammation. For example, the probiotic *L. reuteri* suppresses inflammation and restores epithelial barrier function in DSS-induced colitis by reducing the number of DC subsets, like CD11b+CD11c+ DC, which play a crucial role in producing inflammatory cytokines, preventing neutrophil recruitment, and enhancing the expression of tight junction proteins, such as zonula occludens (ZO-1), and heat shock proteins (HSPs), such as HSP70 and HSP25, in the colon [28]. Treatment of the colon cell line, HT-29, which is induced by LPSs, with *L. reuteri* I5007 showed immunosuppressive activity. Further investigation on experimental animals supported the role of *L. reuteri* I5007 in managing colitis by restoring the number of goblet cells and increasing the expression of MUC-2 [80].

*A. muciniphila* is a mucin-degrading bacterium that is widely known for its effect in maintaining the health of intestinal mucosa. The main role of *A. muciniphila* in IBD is related to its ability to protect against IBD-related intestinal damage by promoting the production of mucin, thus enhancing mucosal barrier strength and reduce gut permeability. The degradation of mucin by *A. muciniphila* produces SCFAs, which are the preferred energy source for goblet cells [95]. Thus, *A. muciniphila* increases mucin-producing goblet cells in the colon and further elevate the expression of *muc1*, *muc5*, and *muc13*, which are genes that encode for mucin production [96]. Moreover, *A. muciniphila* accelerates the normalization of dysbiosis and regulates immune and inflammatory responses [97]. A study on mice with chronic colitis revealed that the immune-modulatory activity and SCFA production of *A. muciniphila* is strain-dependent [7]. Of interest is *A. muciniphila*’s ability to reduce both serum and colon tissue pro-inflammatory cytokines besides lowering neutrophils and inflammatory immune responses mediated by T cells and DC [97,98]. Moreover, the mRNA expressions of antimicrobial peptides, such as Reg3γ and Cramp, were elevated following the treatment of *A. muciniphila* in *C. rodentium* induced colitis [96].

Another important key player probiotic in IBD treatment is *F. prausnitzii*. This species has anti-inflammatory activity; one example is its protective effect against colitis in vitro and in vivo. One study found that the anti-inflammatory activity of *F. prausnitzii* was comparable to a recombinant strain of *L. lactis* that provided the local secretion of IL-10 and restored T cell levels in both acute and chronic chemically induced colitis models [99]. Moreover, *F. prausnitzii* reduced gut permeability and restored intestinal epithelial barrier function [100]. Recent study found that *F. prausnitzii* triggers IL-27 secretion in the TLR6/2-JNK pathway, which subsequently upregulates CD39, leading to increased IL-10 secretion [14]. *Bacteroidetes* is another taxa that is implicated in IBD and has been investigated for treating UC [101]. *B. thetaiotaomicron* has shown a protective effect against colitis through the secretion of pirin-like protein, which reduces the pro-inflammatory NF-κB signaling pathway [102].

The use of probiotic cocktails that contain defined microbial consortia have been also used in IBD treatment. For instance, VSL#3 is a commercially available probiotic composed of eight different strains, *L. acidophilus*, *L. bulgaricus*, *L. casei*, *L. plantarum*, *B. brevis*, *B. infantis*, *B. longum*, and *Streptococcus salivarius* ssp thermophilus. Data reveals that VSL#3 activity is comparable to fecal microbial transplantation (FMT) in UC and is even more safe [103]. VSL#3 increases the levels of SCFAs and anti-inflammatory cytokines while decreasing the production of pro-inflammatory cytokines IL-1β, TNFα, and IFNγ [104]. VSL#3 regulates the differentiation of T follicular helper (Tfh) cells, a subset of CD4+ T helper cells, through its effect on Bcl6 cells, the main transcription factor of Tfh cells. The reduction in Tfh cells resulted in less secretion of IL-21, IgM, IgG, and IgA in mice with DSS-induced colitis [81]. Another probiotic cocktail composed of nine *Lactobacillus* and five *Bifidobacterium* species initiated remission in UC patients after 6 weeks of administration. This effect correlated with a reduction in C-reactive protein (CRP) and an elevation in IgA and in IL-10 production [18]. Another multi-strain probiotic composed of different *Lactobacillus* and *Enterococcus* species had no significant effect on quality of life scores or laboratory values. However, the tested probiotic did reduce the levels of fecal calprotectin significantly, indicating the anti-inflammatory activity of the probiotic in UC patients [83]. A probiotic cocktail composed of *B. infantis*, *L. acidophilus*, *E. faecalis*, and *Bacillus cereus* managed to restore microbial diversity in IBD and enhanced the integrity of mucus and the epithelial layer by promoting the expression of tight junction proteins including occludin, claudin-1, and ZO-1 [105].

Despite the widespread use of probiotics in various aspects of human diseases, many challenges and considerations have not been addressed yet. Research with more precise methodologies and result interpretations is needed to study the long-term and post-market safety of probiotic strains, especially those that are newly identified or isolated. The importance of long-term safety data is particularly crucial for certain populations, such as the immunocompromised, elderly, and newborns, as they are more susceptible to side effects resulting from microbial alterations caused by live therapeutics. Furthermore, the assessment of the horizontal transfer of antibiotic resistance genes and other microbial mutations indeed raises significant concerns about the use of probiotics. Studies have suggested full genome sequence as a valuable tool to assess probiotic safety, identify genes of concern, and determine the potential of probiotics to alter the metabolism of co-administered drugs [106].

**Table 1:** Probiotics in treating IBD.

Probiotic Name/Strains	Dosage Regimen	Outcomes	Model/Study Design	Ref.
Mutaflor^®^ (*E. coli* Nissle 1917)	One capsule (2.5–25 × 10^9^ CFU)/day from day 1 to day 4, then two capsules/day until the end of the study.	-EcN provides significantly equivalent efficacy as mesalazine in preventing relapses of UC.	Randomized, double-blind, double-dummy trial	[87]
*B. longum* 5^1A^	1 × 10^8^ CFU/day from day 0 to day 17 or from day 10 to day 17	-↓ MPO. -↓ IL-1.	DSS-induced colitis mouse model	[91]
*B. longum CECT 7894*	200 μL (5 × 10^8^ CFU)/day for 5 day	-↑ *Bifidobacterium*, *Blautia*, *Butyricicoccus*, *Clostridium*, *Coprococcus*, *Gemmiger*, and *Parabacterioides*. -↓ *Acinetobacter*, *Enterococcus*, and *Pseudomonas*. -↑ Fecal secondary bile acid. -↓ Loss of goblet cells.	DSS-induced colitis mouse model	[94]
*L. reuteri ATCC PTA 4659*	1 × 10^8^ CFU/day for 14 days	-↓ Pro-inflammatory cytokines. -↑ HSP and tight junction proteins. -↓ Neutrophil recruitment.	DSS-induced colitis mouse model	[28]
*L. reuteri* I5007	2 × 10^8^ CFU/day for 14 days	-↓Pro-inflammatory cytokines and ↑ IL-10. -↓ Weight loss and colon length reduction. -↑ MUC-2 expression. -↑Metabolic and biosynthesis pathways.	DSS-induced colitis mouse model	[80]
*L. fermentum* KBL374 and KBL375	1 × 10^9^ CFU/day for 8 days	-↓ Colon shortening, weight loss, DAI score, crypts damage, and goblet cell loss. -↓ Leukocyte infiltration, CCL2, CXCL1, IL-2, IL-4, IL-13, and IFN-γ. -↑ IL-10 and Treg cells. -↑ *Akkermansia* and *Lactobacillus* abundance.	-Peripheral blood mononuclear cells. -DSS-induced colitis mouse model.	[107]
*A. muciniphila*	2 × 10^8^ CFU/day for 56 days	-↓ Pro-inflammatory mediators (e.g., IL-8). -↓ Spleen weight, colon inflammation index, and colon histological score. -↓ Fecal lipocalin-2. -↑ SCFA levels.	HT-29 cells and DSS-induced colitis mouse model	[7]
	1 × 10^8^ CFU for 19 days	-↑ Expression of muc1, muc5, and muc13. -↑ mRNA expressions of Reg3γ and CRAMP.	*C. rodentium* infection-induced colitis	[96]
	3 × 10^9^ CFU/day for 14 days	- ↓ Mucosal barrier damage. -↓ Systemic and colonic inflammatory cytokines. -Improved dysbiosis.	DSS-induced colitis mouse model	[98]
*F. prausnitzii* A2-165	1 × 10^9^ CFU for 1 week	-↓ Microscopic, macroscopic, and histological scores. -↓ MPO. -↓ Weight loss. -↑ IL-10. -↓ IL-12p70, IL-6, and IFN-α. -Restoration of T cell levels.	DNBS-induced colitis mouse model	[99]
	1 × 10^9^ CFU for 10 days	-↓ Intestinal permeability. -Regulation of claudin-4 and F11r expression. -↓ Colonic IL-6, IFN-γ, and IL-4. -↓ Serum IL-6 and IL-22. -↓ Colonic serotonin levels.	DNBS-induced colitis mouse model	[100]
	3 × 10^9^ CFU/day for 14 days	-↓ Mucosal barrier damage. -↓ Systemic and colonic inflammatory cytokines. -Improved dysbiosis	DSS-induced colitis mouse model	[98]
Probiotic 10 billion active cells^®^ (*B. animalis* subsp. lactis, *L. paracasei*, *B. breve*, *L. gasseri*, *L. rhamnosus* (UALr-18), *L. rhamnosus* (UALr-06), *L. acidophilus*, *L. plantarum*, *B. longum*, *B. bifidum*, *L. casei*, *L. reuteri*, *L. lactis*, *B. longum* subsp. infantis)	Three capsules (10^10^/capsule) daily for 6 weeks	-↓ IgA, CRP, PMS, stool frequency, global assessment. -↑ IL-10, RBC, hemoglobin, and hematocrit.	RCT	[18]
Symprove™ (*L. rhamnosus* NCIMB 30174, *L. plantarum* NCIMB 30173, *L. acidophilus* NCIMB 30175 and *E. faecium* NCIMB 30176)	1 mL/kg/day for 4 weeks	-↓ Calprotectin levels. - No significant improvement in quality of life or laboratory tests.	RCT	[83]
VSL #3^®^ (*L. acidophilus*, *L. plantarum*, *L. casei*, *L. delbrueckii* subspecies bulgaricus, *B. breve*, *B. longum*, *B. infantis* and *S. salivarius* subspecies thermophiles)	2.25 × 10^9^ CFU/day for 15 days	-↓ ROS production by peritoneal macrophages. -↓ Basal colonic pro-inflammatory cytokine levels - Improved epithelial barrier function.	*Muc2*^−/−^ mice and DSS-induced colitis mouse model	[104]
	3 × 10^9^ CFU every other day for 60 days	-↓DAI score, HAI score, and MPO activity. -↓ IgM, IgG, IgA, and Tfh cells.	DSS-induced colitis mouse model	[81]
P–qua^®^ (*B. infantis*, *L. acidophilus*, *E. faecalis* and aerobic *B. cereus)*	1.5 × 10^9^ CFU for anaerobic mixture and 0.5 × 10^8^ CFU for aerobic strain	-Improved mucus and epithelial layer function. -↑ Occludin, claudin-1 and ZO-1. -↑ *Bifidobacterium*, *Akkermansia*, *Lactobacillus* and *Bacteroides* abundance.	DSS-induced colitis mouse model	[105]
Mil–Mil^®^ (*B. breve* strain Yakult and *L. acidophilus*)	1 × 10^10^ CFU of *B. breve* + 1 × 10^9^ CFU of *L. acidophilus* once daily for 48 weeks	No significant difference between probiotic and placebo in maintaining relapse-free survival.	RCT	[108]
Biotop capsule^®^ *(L. acidophilus*, *C. butyricum* TO-A, *B. mesentericus* TO-A, and *S. faecalis* T-110)	One capsule three times/day for 1 month	-↓ Stool frequency and Bristol score. -↑ SIBDQ scores.	Human observational study	[109]

MPO = myeloperoxidase, HSP = heat shock protein, ROS = reactive oxygen species, DAI = Disease Activity Index, HAI = histological activity index, SIBDQ = Short Inflammatory Bowel Disease Questionnaire, CRP = C-reactive protein, PMS = Partial Mayo Score, RBC = red blood cell, RCT = randomized clinical trial. DNBS = dinitrobenzene sulfonic acid.

## 3. Prebiotics

Prebiotics are non-digestible fibers that selectively influence the growth and abundance of microbial species that ferment these substrates and produce SCFAs [110]. Commonly used prebiotics include Galacto-oligosaccharide (GOS), short- and long-chain fructans (Fructo-oligosaccharides (FOSs) and inulin), and lactulose. However more investigation is required to ensure the efficacy and safety of using fibers in IBD patients who experience a diminished level of fiber-fermenting microbes and might exhibit an immune reaction towards fiber, as shown in some preliminary studies in mice [111]. A study showed that the β-fructan fibers were only fermented by microbes form healthy subjects or IBD patients in the remission or mild state coupled with reduced secretion of pro-inflammatory cytokines. This action was not observed in microbes of IBD patients [112].

Prebiotics, particularly dietary fibers, have been the subject of several studies regarding their beneficial effects on IBD symptoms and inflammation. Oral inulin produces anti-inflammatory activity, modulates colonic luminal pH, and improves inflammation indicators such as prostaglandin E2, thromboxane B2, and leukotriene B4. However, these anti-inflammatory activities were not observed with fecal inulin enema administration [113]. Oligofructose-enriched inulin (OF-IN) (15 g/day) reduced colitis in active UC patients. This activity is correlated with a significant elevation in butyrate production [114] and enrichment in *B. longum* in CD [115]. Another randomized clinical trial (RCT) showed that 1-kestose, an FOS composed of glucose and fructose, reduced disease activity in UC patients and induced clinical remission. However, this prebiotic reduced the α-diversity among the treated group with no observed changes in SCFA levels [116]. A six-week intake of GOS resulted in a higher abundance of *Bifidobacterium* and *Christenellaceae* only in patients with baseline remission. However, there was no evidence of any effect of GOS on immune modulation, levels of SCFAs, or fecal calprotectin [117]. Moreover, GOS mediated regulatory activity over colitis-associated miRNAs. The beneficial effect of GOS on colon epithelial cells occurs by upregulating the expression of miR-19b, miR-590-5p, and miR-495 and downregulating the expression of miR-29a, miR-31, and miR-142-5p, with the highest effect on miR-19b [118].

Oligosaccharides *obtained from the algae Gracilaria fisheri* (GFO) restored colonic motility, normal weight, and SCFA levels, particularly butyrate [119]. Another type of dietary prebiotic is human milk oligosaccharide (HMO). Being the first prebiotic that is consumed by breastfeeding infants, HMO is associated with various effects on the GI system through its role in early microbiome maturation and immune system modulation in early life [120]. In inflammatory conditions, such as IBD, a pilot clinical trial reported that HMO improved the levels of butyrogenic bacteria such as *Bifidobacterium* and *F. prausnitzii* and further improved quality of life among UC patients [121]. Prebiotics not only promote the growth of the species that directly consume them but also affect the abundance and activity of other commensals that could exert anti-inflammatory activity. For instance, *B. thetaiotaomicron* metabolizes HMOs and lactose, producing intermediate metabolites, such as monosaccharides, acetate, and d-lactate, that cross-feed *Anaerostipes caccae*, a butyrate-producing bacteria [122].

By analyzing the carbohydrate metabolism of metagenome-assembled genomes recovered from the microbiota of patients with Crohn’s disease, several glycosidase enzymes that act on pectin chains were identified from different microbiome species such as *A. muciniphila*, *Barnesiella viscericola* DSM 18177, and *Paraprevotella xylaniphila* YIT 11841. This activity promotes the growth of species with important metabolic roles, enabling them to support other beneficial species that are frequently diminished in CD [123]. Furthermore, pectins from different dietary sources regulate the immune system and alleviate inflammation in colitis models [124]. Resistant starch (RS) is another type of dietary fiber that is associated with a protective effect against colitis. A systematic review and meta-analysis concluded that RS was effective in reducing inflammation-induced damage and promoting clinical remission in IBD patients. This activity was mainly mediated by an elevation in SCFA concentration and modulation of inflammatory mediators and cytokines [125]. A study by Wang et al. showed that RS improved the *Firmicutes*-to-*Bacteroidetes* ratio and supported the growth and activity of health-promoting, non-pathogenic microorganisms such as *Bifidobacterium* and *Lactobacillus* [126].

Polyphenols, such as flavonoids, are a class of natural bioactive compounds that possess anti-inflammatory and immunomodulatory properties. They also benefit gut microflora balance and strengthen the intestinal barrier. Importantly, the anti-inflammatory activity of flavone glycosides, particularly those extracted from *Abelmoschus manihot* flowers, is comparable to steroid administration [127]. Ellagic acid and ellagitannins are polyphenolics that induce the expression of miR-145 and attenuate colitis via miR-145/p70S6K/HIF1α axis. Both p70S6K1 and HIF1α are crucial molecular regulators of inflammation and colitis severity [128]. Polyphenol-rich cranberry enriches the abundance of *Akkermansia* spp. in the gut and prevents intestinal inflammation [129]. Moreover, whole cranberry powder helps correct α-diversity in DSS-treated mice by elevating *Lactobacillus* and *Bifidobacterium* populations and reducing the abundance of potentially harmful bacteria such as *Sutterella* and *Bilophila* [129]. Besides enriching the gut with probiotic species, flavonols such as galangin, quercetin, and fisetin promote the anti-inflammatory function of the microbiome. These prebiotics elevate the production of inflammatory suppressants by *B. adolescentis* that blunt the activity of nitric oxide in LPS-stimulated RAW 264 cells [130].

While prebiotics provide a safe and promising option for treating IBD when investigated in preclinical models, as shown in Table 2, further assessment by large-scale human studies is needed. This is particularly important because of the differences between animals and humans in anatomical and microbial composition. Moreover, many studies have reported that differences in prebiotic structure, molecular size, source, and extraction methods can be translated into differences in activity [124,131]. Furthermore, more clinical trials are required to generate solid scientific evidence regarding the impact of prebiotics on not only clinical outcomes but also on other biochemical markers and microbiological profiles in IBD patients. In previous years, advanced predictive computational approaches have been developed to study the physical, chemical, and structural properties of different prebiotic fibers and their impact on the gut microbiota. For example, a novel machine learning-based computational framework was developed to predict the prebiotic potential of vegetable by-products. This framework aims to rationally identify the most suitable prebiotics that selectively target a defined microbial group. Their findings showed comparable results with evidence obtained from experimental studies [132].

## 4. Synbiotics

Synbiotics are a synergistic mixture of probiotic strains and prebiotic substrates that selectively enhance the growth, colonization, and metabolism of host microorganisms [136]. Synbiotics are divided into two subtypes. While the probiotics and prebiotics in (1) complementary synbiotics provide health benefits without necessarily co-dependent functions, the second type of synbiotics, (2) synergistic synbiotics, requires a prebiotic substrate/s that is selectively utilized by the co-administered probiotic strain/s [136].

Studies reported that synbiotics have a superior effect as anti-inflammatory activity in comparison with probiotics only [137,138]. According to a recent meta-analysis, synbiotics were suggested as adjunctive therapy for the treatment of IBD [139]. Besides the anti-inflammatory properties of synbiotics, the later hold other benefits such as modulating the immune system, restoring microbial balance, and inducing remission. Common synbiotics that target IBD consist of different strains of lactobacilli, *Streptococcus*, and *bifidobacteria* combined with variable doses of dietary fibers like oligosaccharides and inulin [136].

For example, synbiotic preparation composed of six probiotic strains with FOS resulted in remission in IBD patients with mild-to-moderate disease activity and significant reduction in CRP and the sedimentation rate [140]. Another clinical trial found that the intake of *B. longum* with an FOS/inulin mix increased the abundance of *Bifidobacteria* in UC-related mucosal biopsies and reduced inflammatory markers and lesions [141]. Synergistic anti-inflammatory activity between *B. infantis* and the prebiotic xylooligosaccharide (XOS) was reported. This synbiotic reduced the NLR family pyrin domain containing 3 (NLRP3) inflammasome mRNA levels and inhibited oxidative stress in colon tissues [138]. One RCT also reported an improvement in quality of life following treatment with a synbiotic containing *B. longum* with psyllium for one month in 120 UC outpatients [142]. There are some commercially available synbiotics to manage IBD such as Lactocare^®^, for which superior response were observed in chronic (more than 5 years) UC patients compared with those with UC for a shorter duration [143]. Examples of synbiotics studied in IBD are shown in Table 3.

## 5. Postbiotics

Postbiotics are inanimate microorganisms or their secreted metabolites/components that exert beneficial activity (Table 4). Postbiotics have safe profiles and lower chances of transferring antibiotic resistance genes, particularly in immunocompromised patients [147].

Microbiome-derived extracellular vesicles (EVs) are lipid bilayer nanoparticles naturally produced by many commensals. These EVs are employed by microbes as cargos of lipids, proteins, nucleic acids, and other bacterial metabolites to facilitate host–microbiome and microbiome–microbiome communication [148]. These EVs have been implicated in treating many diseases, including IBD, because of their ability to diffuse through cells, which ensures the delivery of therapeutic EV contents directly to target tissues [148].

EVs derived from *C. butyricum* MIYAIRI II 588 reverse gut dysbiosis, mucus layer damage, pro-inflammatory gene expression, and other related microbial functions that are elevated in colitis such as bacterial penetration into epithelial cells and pathogenic *E. coli* infection. Furthermore, these vesicles increase the level of SCFA producers such as *Roseburia* in the gut and restore miR-199a-3p expression [149]. In addition, *C. butyricum*-derived EVs regulate the M1/M2 macrophage balance, promoting a shift towards the anti-inflammatory M2 macrophage status [150].

EVs obtained from *B. acidifaciens* show positive therapeutic results in alleviating colitis by reducing inflammation and enhancing the repair of mucosal damage. Moreover, proteomics analysis of these EVs reveled that they are enriched with various antimicrobial-related proteins that may correspond to colonization resistance activity [151]. Similarly, *A. muciniphila*-derived EVs reduce intestinal permeability by increasing MUC2 expression and rebalance the gut microbiome by enriching *Firmicutes* and reducing *Proteobacteria* abundance [152]. Moreover, both pasteurized *A. muciniphila* and its outer membrane protein, Amuc_1100, decrease colonic infiltration of macrophages and delay colitis-related tumorigenesis by inhibiting DNA breaks and cell apoptosis [153]. Amuc_1100 demonstrates anti-inflammatory activity through TLR2- and TLR4-signaling, which elevates IL-10. Interestingly, one study found that this effect was comparable to the activity of *F. prausnitzii* A2-165 and *L. plantarum* WCFS1, two common probiotics known for inflammatory amelioration [154]. An MS-based proteomics analysis revealed that *A. muciniphila* secrete P9, a protein that elevates the number of anti-inflammatory M2 macrophages [155].

Besides EVs, freeze-dried and spray-dried postbiotics derived from *Saccharomyces boulardii* showed better anti-inflammatory and immune-modulating activity compared with live yeast from the same species (probiotic) [156]. Similarly, non-viable *B. adolescentis* B8589 powder, but not the live probiotic, regulated the gut microbiota β-diversity composition and function when both were tested in DSS-colitis mice [157]. Further research is required to understand the underlying mechanisms by which non-living commensals provide protective benefits to the colon. This may be achieved by utilizing rapidly developing computational proteomics tools and machine learning to study the functional proteins that contribute to the relief of IBD.

Bacterial cell components also serve as another type of postbiotics for treating IBD. The capsular polysaccharide of *B. fragilis* (TP2) showed promising clinical activity in reducing inflammation, ulcer size, and degree of intestinal adhesions when given to enteritis mice model. Furthermore, pharmacokinetics studies of TP2 demonstrated its ability to resist degradation when passing through gastric, intestinal, and colonic conditions [158]. Bacterial flagellins are another major structural component of bacterial flagella that have been proposed as a biological agent for treating colitis. *R. intestinalis* flagellin alleviates inflammation via miR-223-3p/NLRP3 signal transduction in macrophages, thus inhibiting the pyroptosis induced by inflammasome activation [159].

Another type of postbiotic involves the bioactive metabolites produced by the microbiome. Examples of these compounds include SCFAs, bile acids, indoles, and other small molecules. Butyrate boosts the gut’s immune system and induces the differentiation of macrophages with potent antimicrobial function [160]. This is mediated by the inhibition of HDAC in immune cells such as macrophages and monocytes by butyrate. Transcriptomic analysis of *F. prausnitzi* culture supernatants revealed that butyrate, produced by *F. prausnitzi*, upregulates *Dact3*, a gene that negatively regulates the Wnt/JNK signaling pathway, and downregulated IL-8 production [161]. Moreover, Quevrain et al. applied peptidomic analysis to *F. prausnitzi* culture supernatant, which led to the identification of microbial anti-inflammatory molecule (MAM), a 15 kDa protein that shows anti-inflammatory action by interfering with the NF-κB pathway in epithelial cells [162]. Another metabolic product of *F. prausnitzii*, salicylic acid, also exhibits anti-inflammatory effects. Similar to butyrate and MAM, salicylic acid inhibits the activation of NF-κB signaling, thus reducing the production of IL-8 [163]. These studies along with others explain the relationship between low proportions of *F. prausnitzi* and inflammatory diseases. Treatment of mice with colitis by an indole derivative, indole-3-lactic acid (ILA), produced by *Lactobacillus* regulated microbial dysbiosis among colitis-mice models. This regulatory effect occurs through the ability of ILA to mediate microbial cross-feeding, in which higher levels of tryptophan-metabolizing bacteria, such as *Roseburia*, *Faecalibacterium*, and *Clostridium*, were observed after ILA treatment. By promoting the production of other indole derivatives, ILA reduces IL-1β and TNF-α gene expression in the colon and enhances mucosal barrier integrity by elevating the expression of E-cadherin and occludin [30].

**Table 4:** Postbiotics in managing IBD.

Strains/Components	Dosage Regimen	Outcomes	Model/Study Design	Ref.
*C. butyricum* MIYAIRI 588	15 μg of EVs/day	-↑ M2 macrophages. -↑ MUC2 and ZO-1. -↑ *Bacteroidales*, *Lactobacillus*, *Roseburia*, and *Verrucomicrobiales*, -↑ miR-199a-3p.	DSS-induced colitis mouse model	[149,150]
EcN and EcoR12	60 µg/mL of EVs	-↓ Serotonin level. -↑ Occludin and ZO-1. -↓ IL-8 and IL-6.	IL-1β-induced inflammation model in Caco-2 cells	[164]
*A. muciniphila*	0.2 mL of EVs/day for 21 days	-↑ MUC and ZO-1 expression. -↓ Pro-inflammatory factors. - Re-established microbial gut balance.	-DSS-induced colitis mouse model. -RAW264.7 cells.	[152]
	Pasteurized *A. muciniphila* (1.5 × 10^8^ CFU) or Amuc_1100 (3 µg)	-↓ Colon infiltrating macrophages. -↓ Cytotoxic T lymphocytes. -↓ Pro-inflammatory cytokines (e.g., TNF-α, IFN-γ, IL-1β, IL-6, IL-18, and IL-33). -Delayed colitis-induced tumorigenesis.	DSS-induced colitis mouse model	[153]
	100 μg of P9 for 8 weeks	-↑ Levels of anti-inflammatory M2 macrophages (CD11b+CD206+).	HFD-fed mice	[155]
*S. boulardii*	Freeze- and spray-dried yeast cells	-↓ Colonic shortening and tissue damage. -↑ Expression of intestinal tight junction protein. -↓ Pro-inflammatory factors (e.g., IL-1β, IL-6, and TNF-α). -↑ IL-10. -Maintained microbial homeostasis in the intestine.	DSS-induced colitis mouse model	[156]
*B. adolescentis B8589*	0.2 mL non-viable bacterial powder in sterile saline (2 × 10^9^ cell/day) for seven days	-↓ DAI scores and shortening of colon. -↓ Mucosal damage, inflammatory cell infiltration, and loss of crypts. -↑ β-diversity but no change in α-diversity. -↑ *B. intestinalis*, *L. animalis*/*murinus*, and *Romboutsia timonensis*. -↓ *B. bacterium* M12, *Muribaculum intestinale*, and *Bacteroidaceae* sp.	DSS-induced colitis mouse model	[157]
*R. intestinalis*	50 mg/kg of flagellin daily from day 0 to 7	-Inhibited the activation of NLRP3 inflammasome. -↓ Pyroptosis. -↓ DAI score, weight loss, splenomegaly, and lymphocyte infiltration. -↓ IL-18, IL-1 β, IL-6 and, TNF-α. -↑ miR-223-3p.	DSS-induced colitis mouse model	[159]
Bacterial peptidoglycan derivative	100 μg of muramyl dipeptide for 3 days	-↑ Autophagy in the colon. -↓ Gut permeability and intestinal cell apoptosis. -↑ E-cadherin and ZO-1. -↓ Colitis complications (e.g., weight loss, DAI score, and intestinal injury). -↓ TNF-α.	-DSS-induced colitis mouse model. -LPS-induced inflammation in Caco-2 cells.	[165]
*F. prausnitzi*	1.25, 2.5, or 5 mg/kg of the capsular polysaccharide for 7 days	-↓ Colon adhesion and ulcer degree score. -↑ *Firmicutes*. -↓ *Proteobacteria*. -No change in SCFA levels.	2,4-dinitrobenzenesulfonic acid-induced enteritis model	[158]
	500 µL of 10% (*v*/*v*) culture supernatant that contains butyrate	-↑ *Dact3*. -↓ IL-8.	TNF-α-activated HT-29 cells	[161]
	1 mL of supernatant, which contains butyrate, concentrated by 5 times	-Balanced Th17/Treg. -↓ IL-17,IL-6. -↑TGF-β.	TNSB-induced colitis mouse model	[21]
Sodium butyrate (Butyrose^®^)	Three capsules (1800 mg)/day for 60 days	-↑ Butyrogenic bacteria abundance (e.g., *Butyricicoccus* and *Lachnospiraceae* spp.). - Improvement in Qol.	Pilot, monocentric, placebo-controlled randomized study	[166]
*Lactobacillus*	Indole-3-lactic acid	-↓ Colonic shortening and weight loss. -Inhibited production of TNF-α and IL-1β in the serum. -↑ *Clostridium*_XlVa and *Lactobacillus*. -↑ IPA and IAA levels. -↑ E-cadherin and occlusion (by microbial cross-feeding).	IL-10^−/−^ mice	[30]

DAI = Disease Activity Index, EcN = *E. coli* Nissle 1917, HFD = high-fat diet, NLRP3 = nucleotide-binding oligomerization segment-like receptor family 3, QoL = quality of life, IPA = indole-3-propionic acid, IAA = indole-3-acetic acid.

## 6. Engineered Probiotics

**Figure 4:**
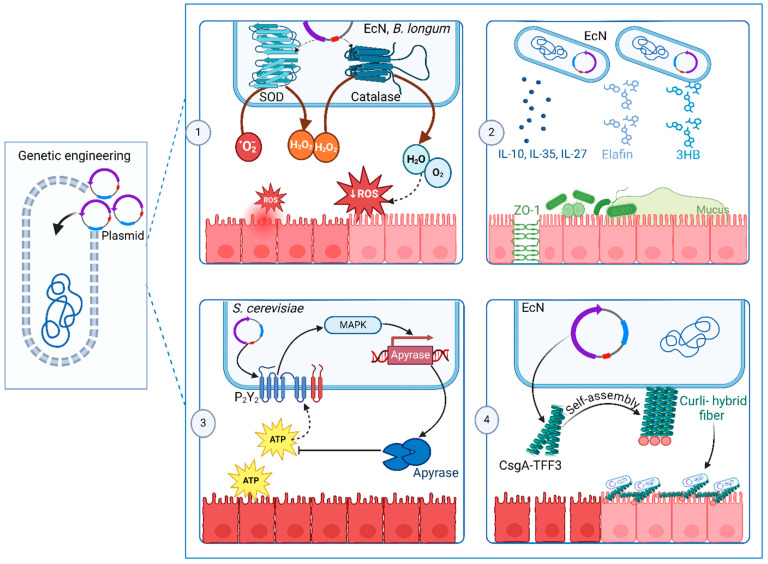
Mechanisms of engineered probiotics in the treatment of IBD. Several microbiome species have been genetically engineered to sense IBD biomarkers and respond by secreting distinct bio-therapeutics. (**1**) *Escherichia Coli* Nissle 1917 (EcN) and *B. longum* were designed to scavenge reactive oxygen species (ROS), thereby reducing oxidative stress by expressing catalase and superoxide dismutase (SOD). These two enzymes degrade ROS and relieve inflammation. (**2**) EcN was also modified to secrete various anti-IBD therapeutics including the ketone (R)-3-hydroxybutyrate (3HB), Elafin, and anti-inflammatory cytokines such as IL-10, IL-35, and IL-27. These molecules can alleviate inflammation, promote barrier integrity, and enhance microbiome diversity. (**3**) Genome editing of *S. cerevisiae* enables the expression of a human P2Y2 purinergic receptor that senses overexpressed ATP levels in inflamed gut regions. Upon ATP detection, P2Y2 triggers the expression of an ATP-degrading enzyme, Apyrase, through the MAPK cascade. This leads to lower levels of ATP and diminished pro-inflammatory cytokine secretion. (**4**) Given the role of barrier dysfunction in IBD, EcN was modified to express CsgA-trefoil factors (TFF3), which self-assemble extracellularly into therapeutic curli hybrid fibers. These fibers can accumulate on the surface of the inflamed colonic mucosa, restoring barrier integrity and preventing bacterial and immune cell translocation.

Modifying symbiotic bacterial strains to deliver and produce certain functional substances or enzymes at specific targeted sites is a new and advanced area of utilizing the microbiome as a therapeutic agent, as demonstrated in Figure 4. Reports of genetically modified probiotics designed to continuously release therapeutics, such as cytokines and therapeutic enzymes, directly to the colon offer a sustainable treatment approach. Recently, Zhou et al. developed genetically engineered EcN probiotics to overexpress two enzymes, i.e., catalase and superoxide dismutase, which function as reactive oxygen species scavengers, consequently relieving inflammation. This engineered bacterium demonstrated its safety and efficiency as an IBD treatment within the DSS-induced murine model. Further, it improved the abundance of butyrate-producing bacteria such as *Lachnospiraceae*_NK4A136 and *Odoribacter* and decreased the abundance of IBD-promoting pathogen *Escherichia-Shigella* [167]. Similarly, another study recently confirmed the development of an engineered *L. casei* that synergistically scavenges ROS and restores gut eubiosis in UC. In mechanistic terms, a viable *L. casei* was embedded with selenium dots in the pericellular film, which was induced by bacteria around its cell wall. Selenium is a natural trace element with antioxidant activity. The addition of selenium not only reduced oxidative stress but also improved the adhesion of the probiotic to the intestinal mucus barrier and provided protection against gastric acid degradation [168].

Cui et al. also reported the development of recombinant IL-10-secreting EcN, providing a disease real-time intervention module that treats UC-related inflammation. Applying tissue-penetrable near-infrared light, which must be converted to blue light by a hydrogel system, initiated the activation of the recombinant EcN in the gut and mediated a controlled release of IL-10 in mice [169]. EcN was also genome-modified to secrete (R)-3-hydroxybutyrate (3HB) through the insertion of components of heterologous 3HB synthesis, where 3HB is a ketone body that serves as an energy supply with some therapeutic activity against a wide range of diseases. It was found that 3HB secreted by the modified orally administrated EcN promoted the levels of gut commensals and SCFAs and further prevented colitis in DSS-induced mice. Improvement in the colon characteristics like weight, length and pro-inflammatory cytokines of the gut tissue were also observed [170]. Based on the previous pieces of evidence supporting the role of Elafin, an endogenous protein that inhibits neutrophil elastase (NE) and proteinase 3, a plasmid-based recombinant EcN encoding for human Elafin protein was developed. The oral administration of this engineered EcN to mice improved colitis-related dysfunctions such as weight loss and colon length shortening besides restoring the levels of tight junction protein ZO-1, beneficial commensals, and SCFAs in colonic tissues to normal [171]. However, the exact mechanism by which Elafin mediates these beneficial effects is still to be investigated.

Recent research has explored the utilization of genetically modified yeast as a therapeutic approach for IBD. Specifically, engineered strains of *S. cerevisiae* were designed to express a modified version of the human P2Y2 purinergic receptor. This receptor is engineered to detect elevated extracellular ATP (eATP) levels, which are associated with inflammatory conditions. Upon sensing elevated eATP levels, the engineered yeast produces apyrase, an enzyme capable of degrading ATP. Extracellular ATP is known to promote the secretion of pro-inflammatory cytokines and inhibit the activity of Treg cells, consequently exacerbating inflammation in IBD. Thus, upon its degradation by the engineered yeast, suppression of intestinal inflammation in IBD-mouse models was reported because of the reduction in the expression of pro-inflammatory cytokines combined with the re-establishment of healthy microbiome composition [172]. Localized delivery is an important consideration when designing a microbial therapeutic agent. Thus, another yeast species, *S. boulardii*, was successfully designed to selectively bind to the inflamed regions within the gut. Mechanistically, *S. boulardii* self-expresses monomeric streptavidin that binds to overexpressed extracellular matrix (ECM) proteins on inflamed colonic tissues. This targeted attachment elevates the resident time of the probiotic in the gut, and consequently, a longer duration of probiotic activity was observed [173].

Besides the secretion of therapeutics, microbiome species have been modified to protect against the initiation and progression of IBD. For example, EcN has been engineered to self-produce fibrous matrices to promote the strength and integrity of gut epithelial. The introduction of a plasmid that encodes a synthetic curli operon capable of producing chimeric CsgA proteins enabled the production of a matrix consisting of curli nanofibers displaying trefoil factors to protect the mucosal layer in the DSS-induced colitis mouse model [174]. Other genome editing studies were performed to enhance the expression of tight junction proteins such as ZO-1, thus regulating intercellular permeability [171]. Table 5 illustrates some examples of engineered probiotics in IBD treatment.

## 7. Fecal Microbial Transplantation

Fecal microbial transplantation (FMT) shows therapeutic efficacy for managing recurrent *Clostridioides difficile* infection. Current clinical evidence highlights the potential of FMT in managing IBD. This approach involves transplanting a defined and highly screened fecal microbial community obtained from a healthy donor to patients. The FMT is administered via nasogastric tube infusion, rectal enemas, colonoscopy, or encapsulated formulations.

A pilot study reported that orally administered FMT capsules reduced fecal calprotectin and improved the colitis index with no changes in α-diversity following FMT [175]. Recent RCT by Costello et al. found that an 8-week administration of pooled FMT resulted in remission in active UC patients [176]. FMT showed a similar rate of remission to prednisone, an oral glucocorticoid commonly used in UC [177]. Interestingly, the high levels of donor intestinal *Candida* and lower eukaryotic viral richness prior to FMT were linked to a more positive response and higher microbial diversity following FMT intervention [178,179]. In addition, remission was associated with the microbial signature of the donor. The enrichment in *Bacteroides* in donor stool was linked to remission in the receiver, while *Streptococcus* species abundance in the donor indicated a failure of FMT in UC patients [180]. However, maintenance of this remission has not been proved within clinical trials. Lahtinen et al. reported that there was no significant difference in the relapse rate between treated (single FMT via colonoscopy) and placebo groups after 12 months of follow-up [181]. Data revealed that FMT improved CD-related symptoms, with 50% of the patients showing steroid-independent remission following FMT [182]. However other studies indicated that FMT did not improve inflammation indicators such as fecal calprotectin or simple endoscopic score in CD patients, while 20% of the patients enrolled in the study experienced adverse events [183].

### 7.1. Role of Gut Bacteria Phages in IBD

Bacteriophage therapy (BT) is an advanced tool that holds therapeutic promise using viruses (phages) that selectively target uncontrolled and undesired bacteria associated with diseases including IBD. In healthy settings, there is a stable and individualized bacteria/phage balance within the gut, in which the abundance or absence of several species is correlated with certain phage populations. For example, Shkoporov et al. found a “persistent personal virome” in which *Microviridae* and crAss-like phages were the most abundant and stable gut phages that linked to *Bacteroides*, *Prevotella*, and *Faecalibacterium* [184]. However, viral metagenomic studies in IBD revealed disturbances to phage levels, with higher levels of active *F. prausnitzii* prophages observed in IBD [185]. This could explain the depletion of *F. prausnitzii* in IBD. Similarly, a heightened prevalence of phages that target *B. uniformis* and *B. thetaiotaomicron* was found in stool samples from IBD patients [186].

Although still in its early stages of development, phage therapy has proven its ability to work as an effective microbiome modulator that influences bacterial community structure without harming the beneficial gut microbe, in contrast to antibiotic treatment. Long-term administration of EcoActive™, a seven-bacteriophage cocktail, selectively targets AIEC strains without triggering dysbiosis in healthy mice and further reduces inflammation symptoms in DSS colitis mice [187]. Of interest, EcoActive is currently in a phase I/IIa RCT to determine the efficacy and safety of this cocktail in patients with CD in remission (ClinicalTrials.gov ID: NCT03808103). A combination of five IBD-causing *Klebsiella pneumoniae* targeted phages successfully prevented inflammation and disease severity in colitis-prone mice [188]. Moreover, phage therapy reduced the levels of pro-inflammatory cytokines such as IL-4 and boosted the members of butyrate-producing *Eubacterium*, according to a recent RCT [189].

Although effective and safe, BT has limitations and challenges that are unaddressed, which delay its transitional clinical application. The majority of studies use PT to target single bacterial strains, which does not mimic the intricate microbial environment associated with the disease. Further concerns relate to the end-products secreted from the lysis of bacteria by the bacteriophage [190]. These released molecules may further elevate inflammation and exacerbate immunity responses. The development of phage-resistant strains has been also reported especially with long-term administration of a single type of PT [191].

### 7.2. Microbiota in the Diagnosis of IBD

Symptoms of IBD are variation-susceptible according to many factors including differences in the level of microbiota dysbiosis that correlate with the disease. Moreover, these symptoms are non-specific and are shared with other diseases, which complicate the process of diagnosis. Other diagnostic tools such as colonoscopy and endoscopy are invasive and sometimes costly, while stool testing, which is commonly used to test the presence or absence of several disease-related chemicals, usually takes time and shows variation in consistency and composition.

Current diagnostics for IBD include invasive techniques such as colonoscopy and endoscopy besides assessing inflammatory biomarkers in fecal and serum samples. Engineered microbes are gaining interest for their potential use in the diagnosis of IBD and cancer [192]. These bacterial sensors are engineered to identify and react with disease biomolecules and yield an output that correlates with the disease. These outputs can be fluorescent, colorimetric, or bioluminescent signals [193].

Engineered microbes were designed to detect IBD by sensing different inflammatory biomarkers. For example, Prindle et al. engineered EcN to sense the presence of calprotectin, the standard laboratory stool biomarker for gut inflammation. Genes that respond to calprotectin were identified through RNA sequencing, and an optimized promoter–luxCDABE cassette system was cloned in EcN. A luminescent signal was generated with sensitivity for calprotectin in vivo and clinical stool samples within 7 days and 12 h, respectively [194]. Nitrate is another chemical that elevates during gut inflammation because of the activation of nitric oxide synthase by cytokines or bacterial LPSs in immune cells and epithelial cells lining the gut mucosa [195]. Woo et al. designed an EcN that can detect nitrate in the colon and fecal samples of mice by introducing a nitrate-responsive genetic circuit to EcN. Upon nitrate detection, green fluorescence output is observed which aids in the diagnosis of colitis [196]. Similarly, other *E. coli* strains were engineered to detect the transiently increased tetrathionate concentrations related to intestinal inflammation, with some of these strains showing a prolonged detection capacity of up to 6 months [197]. A recent study by Zou et al. reported the use of a new probiotic system called intelligent responsive bacteria for diagnosis and therapy (i-ROBOT). This probiotic is composed of EcN with thiosulfate-responsive genetic circuits that assist in IBD diagnosis, continuous monitoring, and amelioration of intestinal inflammation. Thiosulfate is another inflammatory biomarker that, upon its detection by i-ROBOT, releases AvCystatin. In addition to the effect of EcN as a probiotic, AvCystatin relieves inflammation by stimulating the secretion of IL-10 and IL-12/23p40 [198].

### 7.3. Gut Microbiome Potential to Advance Precision Medicine in Managing IBD

The impact of the microbiome on advances in precision medicine is gaining momentum, especially with recent innovations in annotating microbiome-secreted chemistry using mass spectrometry-based metabolomics [199]. A recent study developed a reverse metabolomic platform, which combines the synthesis of small molecules, searching repositories with tandem mass spectrometry, and bioinformatics analysis to reveal specific functional molecules from the microbiome and decipher their association with diseases [200]. Enabled by this platform, Gentry et al. identified a unique microbiome-based metabolomics signature in IBD with the potential to serve as a diagnostic biomarker or therapeutic target. Of interest is the identification of novel bile amidate molecules that are strongly linked to IBD pathology, a link that has been validated across multiple cohort studies. Interestingly, these molecules were increased in patients with Crohn’s disease, particularly during active symptoms. However, this elevation was not observed in patients with UC. Further investigation on the mechanistic underpinning of these associations revealed that some of these amidates dysregulate T cell function and upregulate the production of IFN-γ in CD4^+^ T cells by up to 6-fold in CD [200]. Another mode of action involves agonism of the pregnane X receptor (PXR). PXR is a bile acid nuclear receptor that plays a role in the transport and metabolism of xeonobitics, and its lower expression is associated with IBD. Of note is that PXR agonists such as rifaximin could be used for the treatment of IBD [201].

### 7.4. Microbiome in Precision Medicine: Challenges and Opportunities

The integration of the microbiome alongside other omics profiling is a crucial advance toward precision medicine, enabling personalized therapy, diagnosis, and better outcomes [10]. Although promising, some challenges need to be addressed for effective integration and better outcomes. First, while the set of human genes incorporated in precision medicine is limited, those related to the microbiome are more complex and might involve multiple microbial networking. Second, the determination of causality requires vigorous validation and informatics approaches to assess the fluctuation in microbial communities over time with disease initiation and progression. Third, the heterogenicity in the microbiome is controlled by host genetic and lifestyle factors [202,203].

Several opportunities arise for controlling the host’s immune response and inflammatory diseases such as IBD by modulating the microbiome. Various studies, including those involving association analyses, animal models, and translational research, have shown that the microbiome’s ability to modulate the immune system and influence inflammation levels in the gut are key drivers of its impact on most diseases [204]. For example, leaked microbial metabolites are known to manipulate the host immune response and influence the production of inflammatory mediators, either negatively or positively. These metabolites include LPSs, bile acid derivatives, lithocholic acid, muricholic acid, tryptophan, and SCFAs, among others [205]. Further evidence supporting this perspective is derived from data on intratumor microbiomes and their impact on cancer progression and response to immunotherapies such as checkpoint inhibitors. This effect is primarily attributed to how these microbes influence the host’s inflammatory response and anti-tumor immunity through the metabolic rewiring of cytotoxic CD8^+^ T cells and the recruitment of natural killer T cells [206,207]. A further study revealed a distinct microbial profile in a patient who responded better to an immunotherapeutic, which was characterized by a significant increase in some species such as *Faecalibacterium* and *Bifidobacterium*. These data support the potential of improving therapy outcomes by editing the microbiome.

Precision editing of the microbiome is the next crucial step to the effective implementation of the microbiome in therapy. An interesting study conducted by Zhu et al. showed the possibility of editing microbial communities to reverse dysbiosis and treat colitis. In their study, the oral administration of tungstate restricted the growth of *Enterobacteriacea* which is expanded in a disease state, restored the balance of gut microbes, and caused a 90% reduction in colitis in experimental animals [208]. Tungstate functioned by substituting molybdenum in the molybdoprotein cofactor, which suppressed molybdenum cofactor-dependent metabolism in bacteria and consequently inhibited the growth of *Enterobacteriaceae* and its associated colitis. Another study showed that it is possible to change the composition of the microbiome through host-related signals. For example, nitrate oxide produced during inflammation induced overgrowth of *Enterobacteriaceae*, while mice deficient in inducible nitric oxide metabolic pathway did not show this dysbiosis [209]. Such studies reveal potential therapeutic targets to edit the microbiome.

## 8. Conclusions and Future Directions

A more in-depth comprehension of the microbiome immune interaction and modulation is a crucial step toward microbiome-based precision medicine. This knowledge will reveal more metabolic pathways that could be targeted to advance personalized therapy by altering the immune response and ultimately managing inflammatory diseases such as IBD. A necessary move is to expand integrated omics analysis and large cohort studies that include microbiomes, metabolomics, and transcriptomics across the world and underrepresented populations and make them publicly available. Another essential step is to implement microbiome profiling before therapy in cases of immune and inflammatory-related diseases. This will help to determine whether the patient could benefit from microbiome modulation to optimize therapeutic outcomes. This modulation might include editing the microbial community by use of metabolic modulators or introducing live products, either natural or programmable. In addition to clinical studies of the microbiome of respondent and non-respondent patients during treatment. Furthermore, the effect of the microbiome on drug metabolism should be considered. Moving forward, the implementation of regular microbiome profiling during routine healthcare visits will help to generate a microbiome database and will serve as early signs of diseases.

## Figures and Tables

**Table 2:** Prebiotics in managing IBD.

Prebiotic	Dosage Regimen	Outcomes	Model/Study Design	Ref.
Lactulose	2% lactulose for 14 weeks	-Inhibited inflammation. -Restoration of intestinal microbiota balance. -↓Pathogen abundance.	AOM/DSS mouse model	[133]
Oligosaccharides from *Gracilaria fisheri*	100, 500 or 1000 mg/kg for 2 weeks	-↓ Colitis symptoms (DAI score, weight loss, and colon shortening). -↓ Inflammation. -Restored colonic motility and contractility. -↓ *Enterobacteria*. -↑ SCFA levels.	Acetic acid-induced colitis model	[119]
Synthetic glycans	1% (*v*/*v*) glycan solutions for 8 days	-Changes in microbiome composition. -↓ Weight loss and diarrhea. -↓ Mucosal inflammation.	DSS-induced colitis mouse model	[75]
Inulin	1% (*w*/*v*) or 0.4 g/day.	-↓ pH in colonic lumen. -↓ Mucosal damage. -↓ Inflammatory mediators (e.g., prostaglandin E_2_, thromboxane B_2_, and leukotriene B_4_). -↓ Colonic MPO. -↑ *Lactobacillus*.	DSS-induced colitis mouse model	[113]
	5% inulin plus 2.5% cellulose for 4 weeks	- Exacerbation of DSS-induced colitis. -↑ Weight loss. -↑ Shortening of the colon. -Remarkable splenomegaly. -↑ Proliferation of HCT116 cells. -↑ Luminal succinate.	-AOM/DSS mouse model. -In vitro cell line assay.	[111]
Orafti^®^ Synergy1 (Oligofructose-enriched inulin)	7.5 or 15 g/day for 9 weeks	-↑ Butyrate production. -↑ *Bifidobacteriaceae* and butyrate-producing *Firmicutes*. -↓ Mayo score and fecal calprotectin.	Pilot exploratory clinical study	[114]
1-kestose	10 g/day for 8 weeks	-Induction of remission. -↓ CAI.	RCT	[116]
GOS	2.8 g/day for 6 weeks	-↑ Proportion of normal stools. -↓ Severity of stool urgency. -↑ *Bifidobacterium* and *Christensenellaceae* (in less severe disease). -↓ *Oscillospira* and *Dialister*. -↑ *Anaerostipes.*	Open-label human study	[117]
	5000 mg/day/kg for 2 weeks	-↑ Expression of miR-19b, miR-590-5p, and miR-495. -↓ Expression of miR-29a, miR-31, and miR-142-5p. -Inhibited LPS-induced injury. - ↓ TNF-α, IFN-γ, and IL-1β.	-Human colon epithelial FHC cells. -Helicobacter hepaticus-induced colitis mouse model.	[118]
Orange pectins	200 mg/kg/day for 21 days	-↑ Levels of *Akkermansia* spp. -↑ SCFAs levels. -↑ Expression of GPR43 and GPR109A. -↑ Mucus secretion. -↓ Inflammatory cytokines (IL-6, TNF-α, and IL-1β).	DSS-induced colitis mouse model	[131]
Resistant starch	100 mg/kg and 300 mg/kg from day 1 to day 24	- Body weight restoration. -↓ Thymus atrophy, colon shortening, and spleen hypertrophy. -↓ Inflammatory cytokines (e.g., IL-6, IL-1β, and TNF-α). -↑ Production of SCFAs. *-*↑ *Firmicutes* and *Bacteroidetes*.	DSS-induced colitis mouse model	[126]
Pomegranate polyphenolics	Around 84.468 mL/day for 2 weeks	-↓ Colonocyte proliferation. -↑ *Ruminococcacea*. -↓ TNF-α and IL-1β. -↓ COX-2 and iNOS.	-LPS-treated human CCD-18Co colon myofibroblastic cells. -DSS-induced colitis mouse model.	[128]
Polyphenol-rich cranberry extract	200 mg/kg for 8 weeks.	-↑ Levels of *Akkermansia* spp. -↓ Expression of NF-κB, COX2, and TNF-α.	HFHS-fed mice	[129]
Total flavone of *Abelmoschus manihot*	62.5 mg/kg or 125 mg/kg for 1 week	-↑ Levels of *A. muciniphila* (with 125 mg/kg dose). -↓ *Tenericutes* and *Proteobacteria*. -↓ Weight loss, colonic shortening, and DAI score. -↑ MUC2, KLF4, and ZO-1 mRNA expression.	DSS-induced colitis mouse model	[127]
Epigallocatechin-3-gallate	50 mg/kg for 3 days	-↑ Levels of *A. muciniphila*. -↑ SCFA levels. -↓ Weight loss, rectal bleeding, colonic shortening, and DAI score. -↓ Inflammatory cell infiltration and mucosal damage. -↓ IL-6 and TNF-α.	DSS-induced colitis mouse model	[134]
Galangin, quercetin, and fisetin	25 µM each	-↑ NO suppressant(s) by *B. adolescentis*	RAW264 cells	[130]
Curcumin	2 g/day for 6 months	-↓ Relapse rates. -Improved CAI and EI. -No side effects were reported.	Double-blinded CT	[135]
2′-FL	2 g twice daily for 6 weeks	-↑ *Bifidobacterium*, *F. prausnitzii*, *Eubacterium rectale*-*Clostridium coccoides* group, and *Atopobium* abundance. -↑ SCFA production, -Improvement in quality of life.	-In vitro batch culture fermentation models. -Open-label pilot trial.	[121]

DAI = Disease Activity Index, MPO = myeloperoxidase, CAI = clinical activity index, LPS = lipopolysaccharide, HFHS = high fat/high sucrose, NO = nitric oxide, EI = endoscopic index, CT = clinical trial, 2′-FL = 2′-Fucosyllactose.

**Table 3:** Synbiotics in managing IBD.

Synbiotic	Dosage Regimen	Outcomes	Model/Study Design	Ref.
NBL Probiotic Optima (*E. faecium*, *L. plantarum*, *S. thermophilus*, *B. lactis*, *L. acidophilus*, *B. longum* with FOS)	Two tablets daily (3 × 10^9^ CFU+ 225 mg prebiotic/tablet) for 8 weeks	-↓ CRP and sedimentation values. -Induction of remission.	RCT	[140]
*B. longum* with FOS/inulin mix.	One capsule twice daily (2 × 10^11^ CFU) + One sachet (6 g prebiotic mix) for four weeks	-↓ Inflammatory cytokines (e.g., TNF-α and IL-1β). -No significant change in IL-10 levels. -↓ Sigmoidoscopy score.	RCT	[141]
*C. butyricum* with chitooligosaccharides	1 × 10^8^ CFU/mL + 200 mg/kg prebiotic for 17 days	-↓ Weight loss, colon shortening, tissue damage, and dysbiosis. -↓ TNF-α, IL-1β, and IL-6. -↓ TLR4 expression, p65, and p38 phosphorylation. -↑ SCFA levels.	DSS-induced colitis mouse model	[144]
Lactocare^®^ (*L. casei*, *L. acidophilus*, *L. rhamnosus*, *L. bulgaricus*, *B. breve*, *B. longum*, *S. thermophiles* with FOS)	One capsule twice daily (1 × 10^9^ CFU + 38.5 mg prebiotic) for 8 weeks	-↓ Simple Clinical Colitis Activity Index. -Higher activity observed in long-duration UC patients.	RCT	[143]
*L. pentosus* A14-6 with GOS	1 × 10^9^ CFU/200 μL/day for 21 days	-↓ Colitis symptoms (e.g., weight loss, DAI score, fecal bleeding score, andcolon shortening). -Reversed histological damage and tight junction proteins loss. -↓ Inflammatory cytokines.	DSS-induced colitis mouse model	[137]
MegaSporebiotic (five spore-forming Bacillus strains) + MegaPrebiotic (FOS, XOS and GOS)	8 × 10^9^ CFU + 3775 mg prebiotic/day	-↑ SCFA production. -↓ Ammonium production. -↑ Butyrogenic bacteria abundance. -↑ *Bacillaceae*, *Actinobacteria*, *Lactobacillaceae*, and *Bifidobacteriaceae levels.*	M-SHIME^®^ with fecal inoculum from different adults	[145]
*L. rhamnosus GG* + tagatose	10^9^ CFU/mL + 25 mg prebiotic every other day for 3 week	-↑ *Bacteroides*, *Lactobacillus*, and *Akkermansia* levels. -↓ Diarrhea Score, weight loss, colon shortening, and intestinal damage. -↓ TNF-α, IL-6, and IL-10.	DSS-induced colitis mouse model	[146]
*B. longum* with psyllium	2× 10^9^ CFU + 8 g prebiotic for 4 weeks	-↑ IBDQ scores. -↓ CRP.	RCT	[142]

CRP = C-reactive protein, NLRP3 = NLR family pyrin domain containing 3, IBDQ = Inflammatory Bowel Disease Questionnaire.

**Table 5:** Engineered probiotics for managing IBD.

Strain/s	Mechanism	Outcomes	Model	Ref.
EcN	Overexpression of catalase and superoxide dismutase	-↓ ROS. -Relieved inflammation. -↑ Beneficiary bacteria. -Restoration of intestinal barrier.	Mice	[167]
	Release of IL-10 in response to light	-Inflammation downregulation and protection of intestinal mucosa against injury.	Mice	[169]
	Release of 3HB	-↑ Colonic SCFA levels by 3.1-fold. -↑ Probiotic species (e.g., *Akkermansia* spp.)	Mice	[170]
	Release of Elafin	-Improved the intestinal epithelial barrier. -Facilitated the alleviation of inflammation. -Remodeled the gut microbial community composition. -↑ SCFA levels.	Mice	[171]
*L. casei*	Insertion of selenium dots in the pericellular film	-↓ Intestinal oxidative stress. -Restored microbiota homeostasis. -↑ Probiotic adhesion and gastric acid resistance.	-Mice. -NHP model.	[168]
Yeast strain BS016	Detection and degradation of elevated inflammatory-associated eATP	-Rebalanced a healthy microbiome. -↓ Expression of pro-inflammatory cytokines. -No fibrosis-associated side effects were reported.	Mice	[172]
*S. boulardii*	Selective delivery to the inflamed gut	-↑ Gut resident time and concentration. -Stimulation of anti-inflammatory cytokines. -↑ SCFA levels.	-Cell lines. -Mice.	[173]

EcN = *E. coli* Nissle 1917; ROS = reactive oxygen species; 3HB = (R)-3-hydroxybutyrate; SCFA = short-chain fatty acid; NHPs = non-human primates.
